# Endocannabinoid Enhancement via MAGL Inhibition in CDKL5 Deficiency: Selective Cellular Benefits and Domain-Specific Functional Effects in Adult *Cdkl5* KO Mice

**DOI:** 10.3390/ijms27062773

**Published:** 2026-03-19

**Authors:** Manuela Loi, Nicola Mottolese, Giorgio Medici, Feliciana Iannibelli, Nicolò Interino, Giulia Candini, Federica Trebbi, Angelica Marina Bove, Jessica Fiori, Stefania Trazzi, Elisabetta Ciani

**Affiliations:** 1 Department of Biomedical and Neuromotor Science, University of Bologna, 40126 Bologna, Italy; manuela.loi3@unibo.it (M.L.); nicola.mottolese2@unibo.it (N.M.); giorgio.medici2@unibo.it (G.M.); feliciana.iannibell2@unibo.it (F.I.); giulia.candini4@unibo.it (G.C.); federica.trebbi3@unibo.it (F.T.); angelicamarina.bove2@unibo.it (A.M.B.); 2 IRCCS Istituto Delle Scienze Neurologiche di Bologna, 40139 Bologna, Italy; nicolo.interino@ausl.bologna.it (N.I.); jessica.fiori@unibo.it (J.F.); 3 Department of Chemistry “G. Ciamician”, University of Bologna, 40126 Bologna, Italy

**Keywords:** CDKL5 deficiency disorder, endocannabinoid system, MAGL inhibition, synaptic maturation and neuroprotection, microglial activation and neuroinflammation

## Abstract

CDKL5 Deficiency Disorder (CDD) is a severe neurodevelopmental encephalopathy characterized by early disruptions of synaptic maturation and network stability, leading to persistent motor, cognitive, and behavioral impairments. Given the role of the endocannabinoid system in synaptic development, neuroinflammation, and neuronal resilience, we investigated whether the sustained enhancement of endogenous 2-arachidonoylglycerol (2-AG) signaling via monoacylglycerol lipase (MAGL) inhibition could mitigate key pathological features in adult *Cdkl5* knockout (KO) mice. Using an intermittent 6-week treatment, the MAGL inhibitor JZL184 robustly increased plasma 2-AG levels, reduced MAGL protein levels, and activated CB1-AKT signaling without evidence of receptor desensitization. Despite this clear pharmacodynamic efficacy, behavioral effects were domain-specific: neither dose ameliorated core behavioral deficits, although the higher dose selectively reduced stereotypic jumping and modestly improved cue-dependent associative memory. At the cellular level, JZL184 induced biologically meaningful effects, partially restoring dendritic spine maturation in the primary somatosensory cortex and increasing neuronal survival in the vulnerable CA1 hippocampal region. In contrast, microglial responses were dose-dependent and divergent, with the lower dose exerting anti-inflammatory effects, while the higher dose increased cortical microglial density and Allograft Inflammatory Factor-1 (AIF-1) expression, suggesting engagement of compensatory or off-target mechanisms. Overall, these findings show that MAGL inhibition activates neuroprotective pathways and ameliorates select structural deficits in adult *Cdkl5* KO mice, but is insufficient to produce broad behavioral recovery, highlighting the domain-specific effects of selective 2-AG enhancement via MAGL inhibition and the need for developmentally informed or multimodal therapeutic strategies in CDD.

## 1. Introduction

Cyclin-dependent kinase-like 5 deficiency disorder (CDD) is a severe developmental and epileptic encephalopathy caused by pathogenic variants in the *CDKL5* gene [1]. Affected individuals present with early-onset drug-resistant seizures, profound cognitive impairment, motor dysfunction and autistic-like behaviors [2,3,4], reflecting the widespread disruption of neuronal maturation, dendritic arborization and synaptic plasticity caused by CDKL5 loss [5,6,7,8,9,10]. Although the molecular functions of CDKL5 are increasingly understood, no disease-modifying therapy is currently available, and conventional antiseizure treatments, even when used in polytherapy, provide only limited and often insufficient long-term benefits [11,12]. These limitations underscore the urgent need for novel therapeutic strategies that directly target the synaptic and network dysfunction at the core of CDD pathophysiology.

In recent years, the endocannabinoid system (ECS) has emerged as a potential modulator of developmental encephalopathies [13,14]. The ECS regulates synaptic homeostasis through the endogenous ligands 2-arachidonoylglycerol (2-AG) and anandamide, their metabolic enzymes, and the cannabinoid receptors CB1 and CB2 [15]. However, ECS signaling extends beyond these canonical components, endocannabinoids and related lipid mediators can also engage additional ECS-related targets, including orphan GPCRs (such as GPR55, GPR18, and GPR119), transient receptor potential (TRP) channels (particularly TRPV1, TRPV2, TRPV3, TRPV4, TRPA1, and TRPM8), nuclear peroxisome proliferator-activated receptors (PPARα, PPARγ, and PPARδ), and serotonin receptors (5-HT1A, 5-HT2A/2C, and 5-HT7), among others [15,16]. Importantly, ECS components are highly expressed during early postnatal development, where they orchestrate processes fundamental for brain maturation [17]. CB1 receptors are particularly abundant in corticohippocampal circuits and play essential roles in axonal growth, interneuron migration, dendritic spine maturation, long-range connectivity and the refinement of excitatory–inhibitory balance [18,19,20]. Perturbations in CB1-dependent signaling during these critical windows produce long-lasting abnormalities in synaptic structure and network excitability, suggesting that ECS dysfunction may contribute to the developmental trajectory of CDD.

Consistent with this hypothesis, recent studies in *Cdkl5* mutant mice demonstrate altered expression of CB1R and other endocannabinoid system (ECS)-related targets, including TRPV1, TRPV2, and GPR55, in both hippocampus and cortex, indicating that CDKL5 deficiency is accompanied by a region-specific dysregulation of cannabinoid-associated signaling pathways [21]. These alterations suggest that multiple components functionally linked to the ECS are disrupted in CDD, potentially contributing to synaptic imbalance and network dysfunction. Together with the growing clinical interest in cannabinoids for pediatric epilepsies, these findings support the broader concept that modulation of cannabinoid-related signaling may influence key aspects of CDD pathophysiology.

Indeed, cannabidiol (CBD) has shown antiseizure efficacy in several developmental epileptic encephalopathies and is increasingly used in CDD [12,22,23]. However, CBD does not act as a direct orthosteric CB1 agonist; rather, it functions as a negative allosteric modulator of CB1 receptors, and its mechanisms of action extend well beyond the endocannabinoid system (ECS), encompassing multiple molecular targets and neuromodulatory pathways. Accordingly, while preclinical studies demonstrate that CBD can ameliorate seizure susceptibility, memory impairments, and social deficits in the *Cdkl5* R59X mouse model [21], its impact on core neurodevelopmental features remains variable, and the specific contribution of endocannabinoid signaling to these effects remains unclear. These observations underscore both the therapeutic relevance of cannabinoid-related pathways and the need to disentangle target-specific mechanisms from broader pleiotropic effects.

Within this context, inhibition of monoacylglycerol lipase (MAGL), the principal degradative enzyme of the endocannabinoid 2-AG [24], represents a complementary and mechanistically distinct strategy to probe the role of ECS signaling in CDD. By selectively elevating endogenous 2-AG levels, the MAGL inhibitor JZL184 robustly enhances CB1-mediated signaling within active synapses, leading to reductions in glutamate release, normalization of synaptic plasticity, and attenuation of neuroinflammatory responses [25]. Unlike phytocannabinoids, which exert broad and non-physiological effects across multiple targets, endogenous 2-AG preferentially activates CB1 receptors within the spatial and temporal constraints of native synaptic transmission. This selectivity provides an opportunity to test whether targeted amplification of 2-AG/CB1 signaling is sufficient to restore specific cellular, synaptic, or circuit-level processes disrupted in CDKL5 deficiency, while minimizing off-target effects.

Given the convergence between CDKL5- and ECS-regulated pathways [21], and considering the role of CB1 in establishing synaptic architecture during early development, we hypothesized that enhancing 2-AG signaling through JZL184 in adult *Cdkl5* knockout (KO) mice could compensate for CB1R dysregulation and potentially rescue selected aspects of the established behavioral and neuroanatomical phenotype [7]. In particular, we reasoned that sustained activation of endogenous cannabinoid signaling might reduce network hyperexcitability, improve synaptic organization, and normalize behavioral domains such as memory, stereotypies and anxiety-like responses. Testing this hypothesis is crucial to determine whether selectively enhancing 2-AG signaling through MAGL inhibition represents a viable therapeutic strategy for CDD. Surprisingly, our findings indicate that JZL184 does not rescue the behavioral deficits observed in *Cdkl5* KO mice, suggesting that selective MAGL inhibition alone may be insufficient to reverse established behavioral alterations associated with Cdkl5 deficiency.

## 2. Results

### 2.1. JZL184 Fails to Rescue Core Behavioral Deficits in Adult Cdkl5 KO Mice, with Only Mild Improvements at the Higher Dose

**Figure 1:**
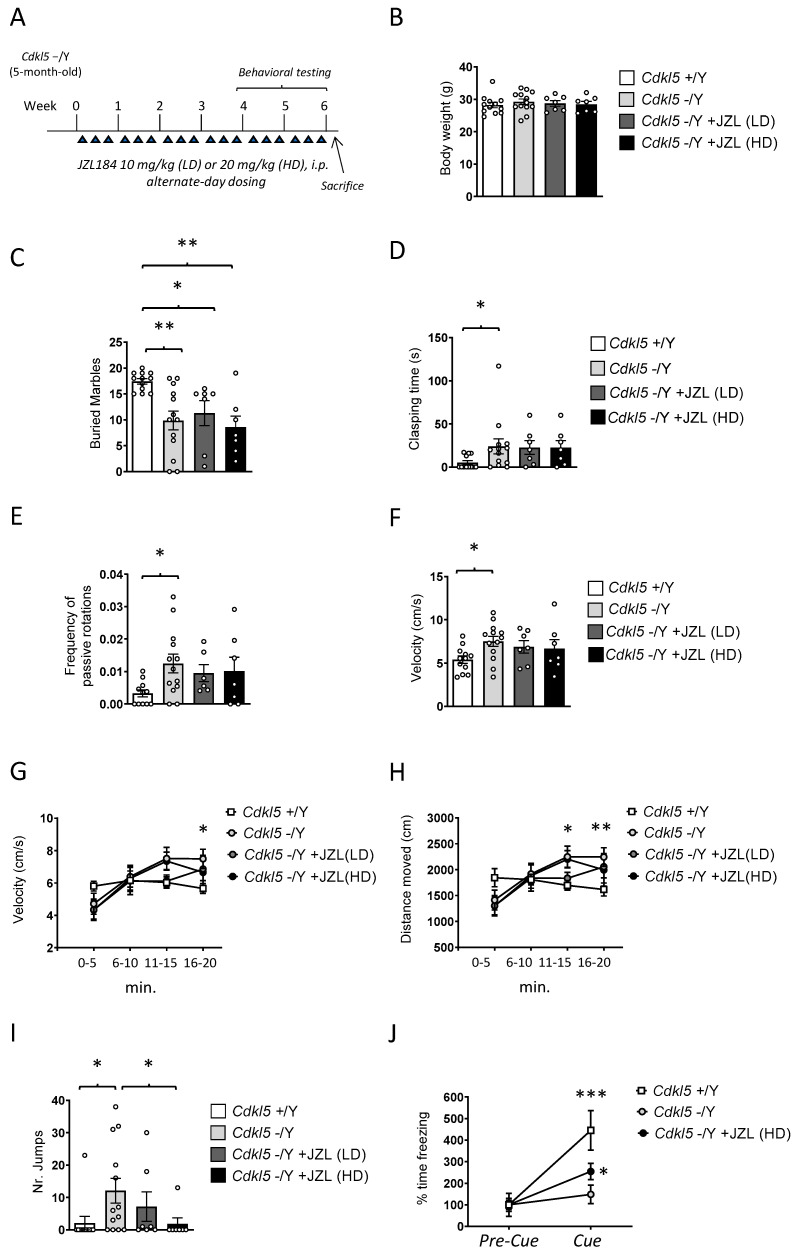
Experimental design and behavioral assessment following 6-week JZL184 treatment. (**A**) Experimental timeline showing the intermittent JZL184 treatment regimen (10 mg/kg Low dose [LD] or 20 mg/kg High dose [HD], i.p., three times per week as indicated by the triangles) administered for 6 weeks starting at 5 months of age. Behavioral testing was conducted during the final two weeks of treatment. (**B**) Body weight in grams measured at the end of the treatment period of vehicle-treated wild-type (+/Y *n* = 12), vehicle-treated *Cdkl5* KO (−/Y *n* = 13) and JZL184-treated *Cdkl5* KO mice (−/Y JZL LD *n* = 7; −/Y JZL HD *n* = 7). (**C**) Autistic-like features in treated *Cdkl5* −/Y mice. Marble-burying performance, expressed as the number of marbles buried after 30 min in the same groups described in (**B**). (**D**) Total hindlimb clasping time measured during a 2-min tail suspension test in vehicle-treated *Cdkl5* +/Y (*n* = 12) and −/Y mice (*n* = 12) as well as in JZL184-treated *Cdkl5* −/Y mice receiving either a low dose (LD, *n* = 7) or a high dose (HD, *n* = 7). (**E**) Rotarod performance expressed as the frequency of passive rotations in vehicle-treated wild-type (+/Y *n* = 11), vehicle-treated *Cdkl5* KO (−/Y *n* = 13) and JZL184-treated *Cdkl5* KO mice (−/Y JZL LD *n* = 6; −/Y JZL HD *n* = 7). (**F**) Locomotor activity assessed in a 20-min open field test. The graph shows the average locomotion velocity in vehicle-treated wild-type (+/Y *n* = 12), vehicle-treated *Cdkl5* KO (−/Y *n* = 13) and JZL184-treated *Cdkl5* KO mice (−/Y JZL LD *n* = 6; −/Y JZL HD *n* = 7). (**G**,**H**) Time-binned analysis of locomotor velocity (**G**) and total distance traveled (**H**) during the 20-min open field session in mice as in (**F**). (**I**) Anxiety-like behavior was assessed based on the number of vertical jumps performed by mice during the open field test. (**J**) Cue-dependent freezing, expressed as the percentage of freezing time during fear conditioning, in vehicle-treated *Cdkl5* +/Y (*n* = 12) and −/Y (*n* = 12) mice and in *Cdkl5* −/Y mice treated with a high dose of JZL184 (HD, *n* = 7). Values are represented as means ± SEM. Statistical analyses were performed using one-way ANOVA (**B**–**F**,**I**) or two-way ANOVA (**G**,**H**,**J**), followed by Fisher’s LSD post hoc test. * *p* < 0.05, ** *p* < 0.01, *** *p* < 0.001.

To evaluate whether sustained enhancement of 2-AG signaling could mitigate behavioral deficits in *Cdkl5* KO mice, JZL184 was administered at two doses (10 or 20 mg/kg, i.p.) on an alternate-day schedule for 6 weeks in adult (5-month-old) *Cdkl5* KO male mice (Figure 1A). Behavioral testing was conducted during the final two weeks of treatment.

Body weight was monitored once per week throughout the 6-week treatment period to evaluate any potential systemic or metabolic consequences of MAGL inhibition. Final body weight measurements, reported in Figure 1B, showed no significant differences between vehicle-treated *Cdkl5* KO mice and those receiving either the 10 mg/kg or 20 mg/kg JZL184 regimens (Figure 1B). Likewise, JZL184-treated animals did not differ from wild-type controls at the end of the study. The stability of body weight across all groups indicates that the intermittent dosing schedule was well tolerated and did not elicit detectable metabolic stress, appetite alterations, or signs of compromised general health. These findings provide confidence that the behavioral and neurobiological outcomes described in the following sections are unlikely to be influenced by treatment-induced malaise or overall declines in wellbeing, thereby supporting the validity of the subsequent analyses.

To evaluate repetitive and perseverative behaviors, we performed the buried marble test. As expected, wild-type (+/Y) mice buried significantly more marbles than *Cdkl5* KO (−/Y) mice (Figure 1C), confirming the previously reported reduction in goal-directed digging [26]. Six-week JZL184 treatment failed to improve this deficit: neither the low dose (10 mg/kg) nor the high dose (20 mg/kg) increased marble-burying performance, and treated mutants behaved similarly to vehicle-treated *Cdkl5* KO mice (Figure 1C).

Clasping behavior (clasping time) was assessed as an index of motor and postural dysfunction. As expected, *Cdkl5* KO mice showed a pronounced clasping phenotype (Figure 1D), with 11 out of 13 animals displaying sustained hindlimb flexion when suspended by the tail. Wild-type mice generally exhibited normal hindlimb extension [7]; however, we unexpectedly observed clasping in 4 out of 12 wild-type animals. This low-penetrance clasping in wild-type mice may reflect stress induced by repeated handling and prolonged manipulation during the treatment regimen, rather than a genuine motor deficit.

Six-week JZL184 treatment did not ameliorate clasping in *Cdkl5* KO mice. Both the low and high doses yielded clasping times comparable to vehicle-treated mutants (Figure 1D), with 6 out of 7 treated animals in each dose group exhibiting clear clasping. These findings indicate that enhanced 2-AG signaling is insufficient to rescue this postural abnormality.

Together, these results indicate that sustained MAGL inhibition and 2-AG elevation are insufficient to rescue either perseverative digging deficits or motor and postural abnormalities in *Cdkl5* KO mice, two robust and widely documented behavioral phenotypes of this model [7,26].

Motor coordination and balance were evaluated using the rotarod test, with particular attention paid to the number of passive rotations, an established indicator of impaired motor control and reduced ability to maintain active footing on the rotating rod. Latency to fall was also recorded; however, no significant differences were observed between groups for this parameter in adult *Cdkl5* KO male mice (Figure S1A). As expected, *Cdkl5* KO mice exhibited a significantly higher number of passive rotations compared with wild-type controls (Figure 1E), confirming the presence of marked deficits in motor coordination and postural stability in this model. Six-week JZL184 administration did not ameliorate these impairments. Neither the low dose (10 mg/kg) nor the high dose (20 mg/kg) produced any measurable improvement in rotarod performance, and JZL184-treated *Cdkl5* KO mice remained indistinguishable from vehicle-treated mutants (Figure 1E).

Locomotor activity was quantified in the open field by measuring both average velocity and total distance traveled across the 20-min trial. Analysis of mean velocity revealed that *Cdkl5* KO mice displayed significantly higher locomotor activity compared with wild-type controls (Figure 1F), consistent with the hyperactive phenotype previously described in this model [7]. Six-week JZL184 treatment produced only minimal effects on this abnormality: although a slight reduction in mean velocity was observed, particularly in the 20 mg/kg group (Figure 1F), the change did not reach statistical significance, and JZL184-treated *Cdkl5* KO mice remained markedly more active than wild-type animals.

A time-binned analysis of locomotion in 5-min intervals revealed clear differences in activity dynamics between genotypes. Wild-type mice maintained a relatively constant running speed across the entire 20-min session (Figure 1G), indicating stable exploratory behavior and normal habituation. In contrast, *Cdkl5* KO mice showed a progressive increase in hyperactivity over time, with significantly elevated velocity during the second half of the trial (Two-way ANOVA, effect of time: *p* < 0.001). The hyperlocomotor phenotype became most pronounced in the 10–20-min interval, when *Cdkl5* KO mice ran substantially faster than wild-type controls (Figure 1G). Treatment with JZL184 produced only modest effects on this temporal profile. Mice receiving either dose showed a slight attenuation of running speed during the final 5-min segment (16–20 min), with the trend more apparent in the 20 mg/kg group (Figure 1G). Although this late-session reduction may suggest a partial normalization of habituation dynamics, the effect remained small and did not reach statistical significance.

Total distance traveled across the 20-min trial mirrored the velocity findings. *Cdkl5* KO mice covered significantly greater distances than wild-type animals (Figure 1H), particularly during the second 10 min of the test, consistent with their heightened hyperactivity. Six-week JZL184 treatment failed to rescue this phenotype (Figure 1H). Both doses produced only minor reductions in distance traveled, again slightly more pronounced at 20 mg/kg, but overall locomotor activity remained markedly elevated in the *Cdkl5* KO groups.

Time spent in the central area of the open field was also analyzed as an index of anxiety-like behavior. No significant differences were detected between genotypes or treatment groups (Figure S1B). A modest increase in center exploration was observed in high-dose JZL184-treated *Cdkl5* KO mice during the final 5-min interval compared with vehicle-treated wild-type and *Cdkl5* KO mice; however, this effect did not reach statistical significance. Overall, central time did not reveal robust genotype- or treatment-dependent differences in adult *Cdkl5* KO male mice.

Stereotypic vertical jumping was quantified as an index of repetitive and disinhibited motor behavior. *Cdkl5* KO mice displayed a markedly elevated jumping phenotype across the 20-min trial, with a substantially higher number of jumps compared with wild-type controls (Figure 1I). This abnormal motor output was also more penetrant: only 1 out of 11 wild-type mice exhibited any jumping, whereas 9 out of 14 *Cdkl5* KO animals displayed clear stereotypic jumping during the session. These results confirm that excessive vertical jumping is a robust behavioral hallmark of CDKL5 deficiency, reflecting dysregulated motor activation and impaired behavioral control.

Six-week JZL184 treatment produced a dose-dependent reduction in this phenotype. The low dose (10 mg/kg) decreased the proportion of jumpers to 2 out of 7 animals and led to a modest reduction in jump counts, although levels remained above those of wild-type mice (Figure 1I). Notably, the high dose (20 mg/kg) markedly suppressed jumping behavior (Figure 1I), with only 1 out of 7 treated mice displaying jumps and with the number of jumps restored to wild-type values. This indicates that stronger enhancement of 2-AG signaling is capable of normalizing this specific stereotyped motor abnormality.

Together, these findings demonstrate that high-dose MAGL inhibition effectively rescues a prominent motor-disinhibition phenotype in *Cdkl5* KO mice, while lower-dose treatment provides only partial benefit.

Passive avoidance was used to assess associative learning and memory. On training day (Day 1), no significant differences in step-through latency were observed among wild-type, *Cdkl5* KO, and JZL184-treated groups (Figure S1C), indicating comparable acquisition of the task. During the passive avoidance retention test (Day 2), no significant differences were detected between wild-type and *Cdkl5* KO mice (Figure S1C). This lack of effect was primarily due to the unexpectedly poor retention exhibited by wild-type mice, which showed little evidence of aversive memory consolidation (mean latency close to vehicle-treated *Cdkl5* KO levels). As a result, the typical “ceiling effect” expected in wild-type animals was absent, masking any potential deficit in *Cdkl5* KO mice and preventing the detection of possible rescue by JZL184 treatment. A similar absence of improvement was observed in JZL184-treated *Cdkl5* KO animals (Figure S1C), whose performance remained comparable to both wild-type and vehicle-treated *Cdkl5* KO mice.

Given the overall lack of discriminatory power of the passive avoidance task in our cohort, and its inability to reveal genotype-related impairments or treatment-dependent rescue effects, we turned to fear conditioning, a paradigm known to be more sensitive and robust for detecting associative learning and memory deficits in mouse models of neurodevelopmental disorders [27].

Fear conditioning was therefore employed as an alternative paradigm to evaluate associative learning and memory. For this experiment, only the high dose of JZL184 (20 mg/kg) was tested, as previous behavioral analyses indicated that the lower dose produced minimal or no effects across multiple domains, whereas the high dose yielded the only detectable behavioral improvements.

On the conditioning day (Day 1), no differences in freezing percentage were observed among the three groups, wild-type, *Cdkl5* KO, and JZL184-treated *Cdkl5* KO, either during the pre-shock period or immediately following shock delivery (Figure S1D). This indicates that baseline freezing behavior and shock responsiveness were comparable across groups.

During the cue-induced recall test on Day 2, freezing levels in the pre-cue phase did not differ among groups (Figure S1E), confirming that baseline immobility was not affected by genotype or treatment. As expected, wild-type mice exhibited a significant increase in freezing from the pre-cue to the cue phase (Figure 1J), reflecting intact cue-dependent associative memory, whereas *Cdkl5* KO mice failed to show a comparable increase (Figure 1J), in line with the well-documented impairment in associative fear memory reported for this model [28].

Importantly, treatment with high-dose JZL184 partially restored this deficit. Although freezing levels during the cue phase did not significantly differ from vehicle-treated *Cdkl5* KO mice, JZL184-treated mutants displayed a significant within-group increase in freezing from pre-cue to cue (Figure 1J), indicating recovery of cue responsiveness. While performance did not reach wild-type levels, these findings suggest that the sustained elevation of 2-AG signaling can selectively restore associative cue processing without fully normalizing overall freezing magnitude.

Mechanical nociceptive thresholds were assessed using the von Frey filament stimulation test, which measures both paw withdrawal latency and force threshold in response to calibrated mechanical pressure and is widely used as an index of basal mechanical pain sensitivity. Across all experimental groups, both withdrawal latency and force threshold were comparable (Figure S1F). Wild-type mice, *Cdkl5* KO mice, and *Cdkl5* KO animals treated with JZL184, at both 10 mg/kg and 20 mg/kg, displayed similar response times to von Frey stimulation (Figure S1F), with no significant differences detected.

These findings indicate that sustained MAGL inhibition does not alter mechanical pain perception and that enhancement of 2-AG signaling fails to exert analgesic or hypoalgesic effects under these conditions. Importantly, the preservation of normal nociceptive responsiveness across groups confirms that the behavioral phenotypes observed in the open field and fear conditioning paradigms are unlikely to be confounded by changes in pain sensitivity or stimulus reactivity.

### 2.2. JZL184 Effectively Inhibits MAGL Activity and Increases 2-AG Levels in Cdkl5 KO Mice

**Figure 2:**
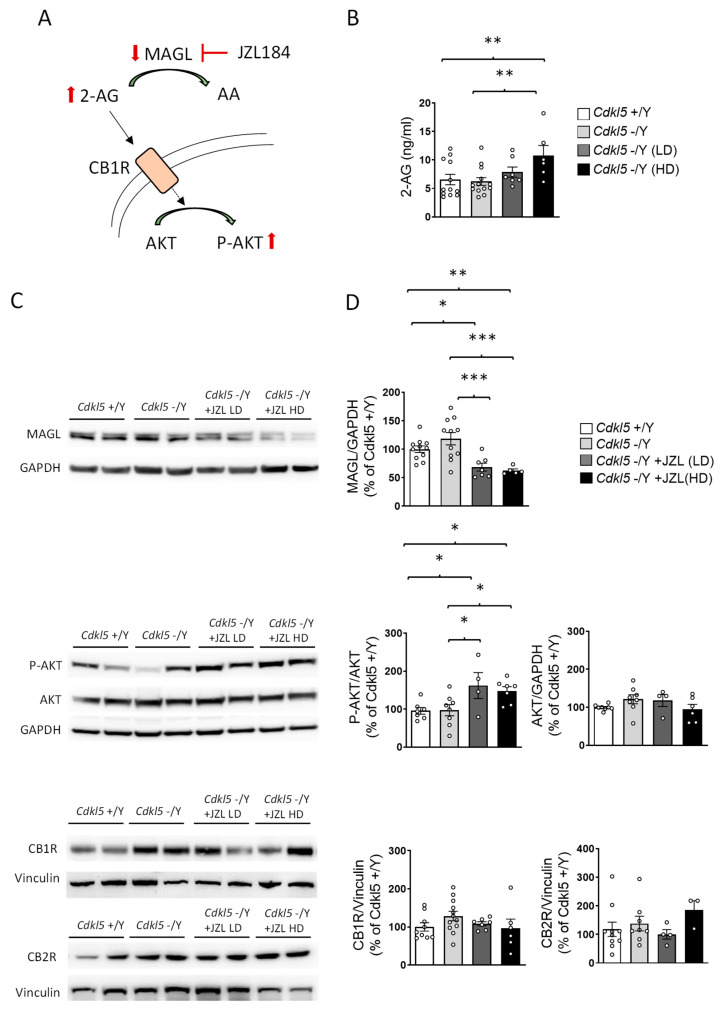
Biochemical assessment of MAGL inhibition and downstream signaling. (**A**) Proposed model of the effects of 6-week JZL184-mediated MAGL inhibition on endocannabinoid signaling and downstream biological outcomes in *Cdkl5* KO mice. Inhibition of MAGL increases 2-AG levels, thereby enhancing CB1R activation and promoting AKT phosphorylation. (**B**) Mass spectrometry-based biochemical analyses of plasma 2-AG levels were performed at the end of the 6-week treatment paradigm in vehicle-treated *Cdkl5* +/Y (*n* = 12) and −/Y (*n* = 13) mice, as well as in JZL184-treated *Cdkl5* −/Y mice receiving either a low dose (LD, *n* = 7) or a high dose (HD, *n* = 6). (**C**,**D**) Western blot analysis of MAGL, CB1R, CB2R, phospho-AKT (Ser473), total AKT, GAPDH and Vinculin protein expression in hippocampal extracts from mice as described in (**B**). Representative immunoblots from two animals per group are shown in panel (**C**). Histograms in (**D**) show quantification of phospho-AKT (Ser473) normalized to total AKT, total AKT and MAGL normalized to GAPDH, and CB1R and CB2R levels normalized to Vinculin. The data are shown as the mean ± SEM and are expressed as a percentage relative to the controls. Statistical significance was assessed using one-way ANOVA followed by Fisher’s LSD post hoc test. * *p* < 0.05, ** *p* < 0.01 and *** *p* < 0.001. Abbreviations: MAGL = Monoacylglycerol Lipase, 2-AG = 2-arachidonoylglycerol, AA = Arachidonic Acid, CB1R = Cannabinoid Receptor Type 1.

To determine whether the behavioral effects described above were supported by effective target engagement (Figure 2A), we performed mass spectrometry-based biochemical analyses on blood samples collected from the same cohort of mice at the conclusion of the 6-week treatment paradigm. Establishing pharmacodynamic engagement is critical for interpreting the behavioral findings, as it demonstrates that the experimental manipulation achieved the intended modulation of the 2-AG/MAGL axis.

After six weeks of treatment, basal plasma 2-AG levels did not differ between vehicle-treated wild-type (+/Y) and *Cdkl5* KO (−/Y) mice (Figure 2B). In *Cdkl5* KO mice, JZL184 treatment increased plasma 2-AG levels compared with vehicle-treated controls (Figure 2B), confirming effective MAGL inhibition. While the low dose (10 mg/kg) produced only a trend toward increased 2-AG levels, the high dose (20 mg/kg) significantly elevated plasma 2-AG levels (Figure 2B).

Western blot analysis of hippocampal lysates showed a marked reduction in MAGL immunoreactivity in both JZL184-treated groups (Figure 2C,D), consistent with effective MAGL blockade reported for this compound in vivo [29,30]. In contrast to plasma 2-AG levels, which were significantly elevated at the high dose, MAGL protein levels did not differ between the two dosing regimens, suggesting similar target engagement at the lower dose, as inferred from MAGL protein reduction and downstream signaling activation, although enzymatic activity was not directly measured. Consistent with this interpretation, phosphorylation of AKT, a downstream readout of CB1 activation, was similarly increased at both doses (Figure 2C,D). This pattern supports the existence of a pharmacodynamic ceiling effect, whereby further increases in JZL184 dose do not translate into additional suppression of MAGL expression or amplification of CB1-AKT signaling.

Importantly, neither CB1 nor CB2 receptor expression differed across groups (Figure 2C,D), indicating that the intermittent dosing schedule prevented receptor downregulation.

Together, these biochemical data demonstrate that JZL184 produced reliable and sustained engagement of its molecular target in the same animals used for behavioral testing. This confirms that the limited behavioral improvements observed are unlikely to reflect insufficient target engagement. Rather, despite robust molecular activation, selective enhancement of 2-AG signaling via MAGL inhibition appears to exert domain-specific effects and is insufficient to fully reverse established behavioral alterations in adult *Cdkl5*-deficient mice.

### 2.3. JZL184 Partially Rescues Cortical Spine Maturation and Hippocampal Neuronal Survival in Cdkl5 KO Mice

Because 2-AG/CB1 signaling has been implicated in activity-dependent structural plasticity and local synaptic remodeling at dendrites [31], cortical dendritic spines provide an appropriate readout to assess whether sustained MAGL inhibition can influence synaptic development in *Cdkl5* KO mice. This is particularly relevant given that CDKL5 deficiency is associated with documented impairments in synaptic maturation [32,33], which are thought to contribute to the behavioral phenotype of the disorder. For these reasons, we focused our analysis on pyramidal neurons in layers II/III of the primary somatosensory cortex (S1), a region where CDKL5 is highly expressed and critically involved in shaping excitatory synaptic architecture [34].

**Figure 3:**
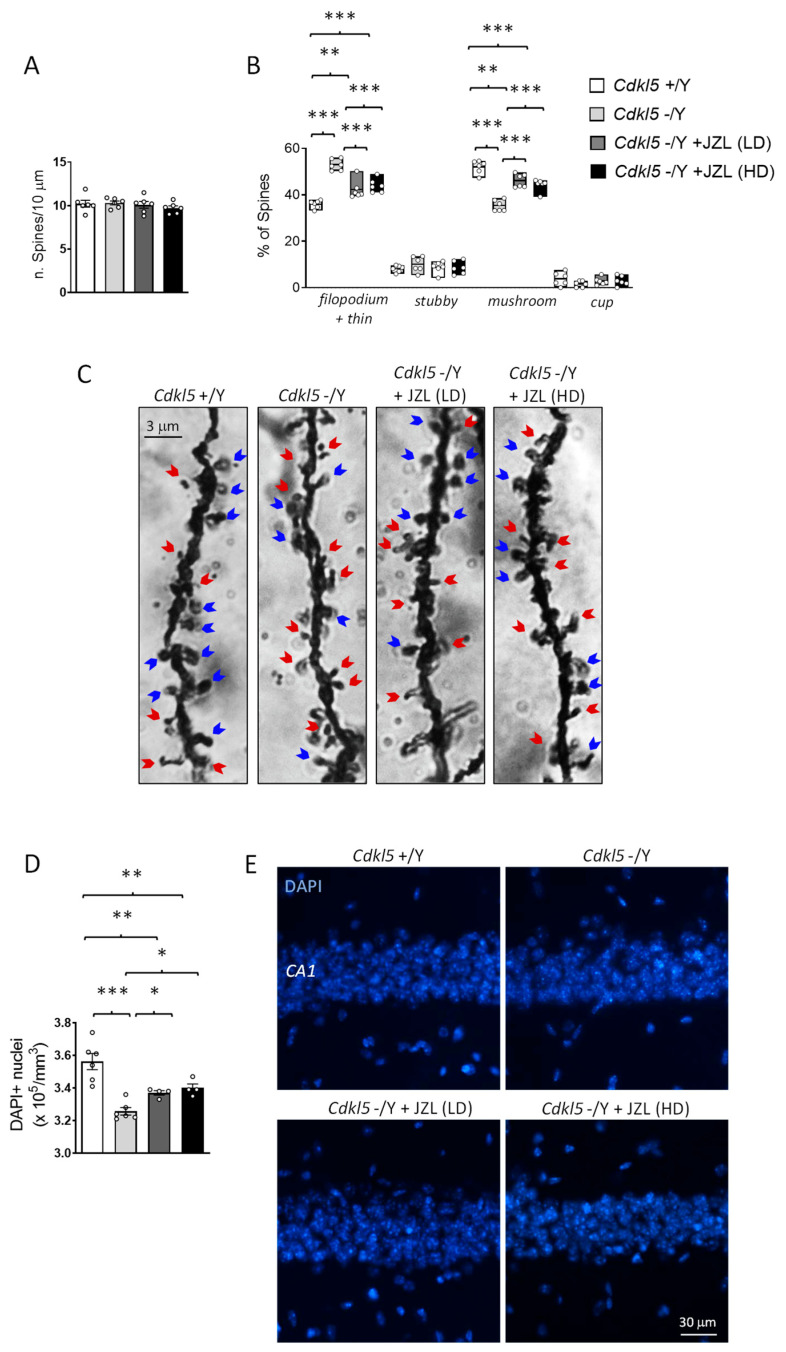
Analysis of dendritic spine morphology and hippocampal neuronal density. (**A**) Total dendritic spine density measured on Golgi-stained basal dendrites of pyramidal neurons located in layers II/III of the primary somatosensory cortex (S1) from vehicle-treated wild-type mice (*n* = 6), vehicle-treated *Cdkl5* KO mice (*n* = 6), and *Cdkl5* KO mice treated with JZL184 at the low dose (LD, 10 mg/kg *n* = 6) or high dose (HD, 20 mg/kg *n* = 6). (**B**) Quantification of dendritic spine morphology showing the relative proportion of immature (filopodia-like and thin) and mature (mushroom- and cup-shaped) spines expressed as a percentage of the total number of protrusions in mice as described in panel (**A**). (**C**) Representative Golgi-stained images of basal dendrites from cortical pyramidal neurons for each experimental group. Blue arrows indicate mature spines, whereas red arrows indicate immature spines. (**D**) Quantification of DAPI-positive nuclei in the CA1 pyramidal layer of the hippocampus from vehicle-treated *Cdkl5* +/Y (*n* = 6) and −/Y (*n* = 6) mice, and JZL184-treated *Cdkl5* −/Y mice receiving either a low dose (LD, *n* = 4) or a high dose (HD, *n* = 4). (**E**) Representative DAPI-stained hippocampal sections from one mouse per group. Data are expressed as mean ± SEM. Statistical significance was determined using one-way ANOVA followed by Fisher’s LSD post hoc test. * *p* < 0.05, ** *p* < 0.01, *** *p* < 0.001.

Golgi staining revealed that overall spine density did not differ between wild-type and *Cdkl5* KO mice (Figure 3A). However, spine classification uncovered a clear alteration in spine morphology: *Cdkl5* KO neurons displayed a higher proportion of immature spine types (filopodia-like and thin spines) and a reduced proportion of mature forms such as mushroom, and cup-shaped spines (Figure 3B,C). This shift toward immature morphologies reflects defective synaptic stabilization and delayed spine maturation, hallmark features of CDKL5-related synaptopathy [5].

Six-week JZL184 treatment partially corrected these abnormalities. Although spine density remained unchanged across all groups (Figure 3A), both the 10 mg/kg and 20 mg/kg regimens shifted the morphological distribution toward a more mature profile (Figure 3B,C). JZL184-treated *Cdkl5* KO neurons exhibited an increased proportion of mushroom spines and a corresponding reduction in filopodia/thin spines relative to vehicle-treated mutants (Figure 3B,C). The rescue, however, was incomplete, as the spine subtype distribution did not fully return to wild-type levels. These findings indicate that sustained enhancement of 2-AG signaling can promote aspects of cortical spine maturation but is insufficient to fully normalize synaptic architecture in the absence of CDKL5.

Because endocannabinoid signaling, particularly 2-AG/CB1 activation, has been implicated in neuronal protection, trophic support, and resistance to cellular stress [19,35], we examined whether sustained MAGL inhibition could mitigate hippocampal vulnerability in *Cdkl5* KO mice. Neuronal survival was quantified by counting DAPI-positive nuclei within the CA1 pyramidal layer, a region known to be structurally and functionally impaired in CDKL5 deficiency and highly relevant for learning and memory [7,26,36].

As expected, *Cdkl5* KO mice displayed a significant reduction in the number of DAPI-positive nuclei in CA1 compared with wild-type controls, consistent with decreased neuronal survival reported in this model [26,37]. Six-week JZL184 treatment partially rescued this deficit: both the 10 mg/kg and 20 mg/kg regimens increased CA1 neuronal counts relative to vehicle-treated *Cdkl5* KO mice. However, the recovery remained incomplete, and neuron numbers did not reach wild-type levels.

These findings indicate that sustained enhancement of 2-AG signaling can confer partial neuroprotection in the hippocampus of *Cdkl5* KO mice, improving neuronal survival but not fully reversing the underlying susceptibility associated with CDKL5 loss. The incomplete rescue suggests that, while MAGL inhibition engages protective pathways, additional mechanisms likely contribute to the hippocampal atrophy observed in this model.

### 2.4. JZL184 Exerts Dose-Dependent and Only Partial Effects on Microglial Activation in Cdkl5 KO Mice

**Figure 4:**
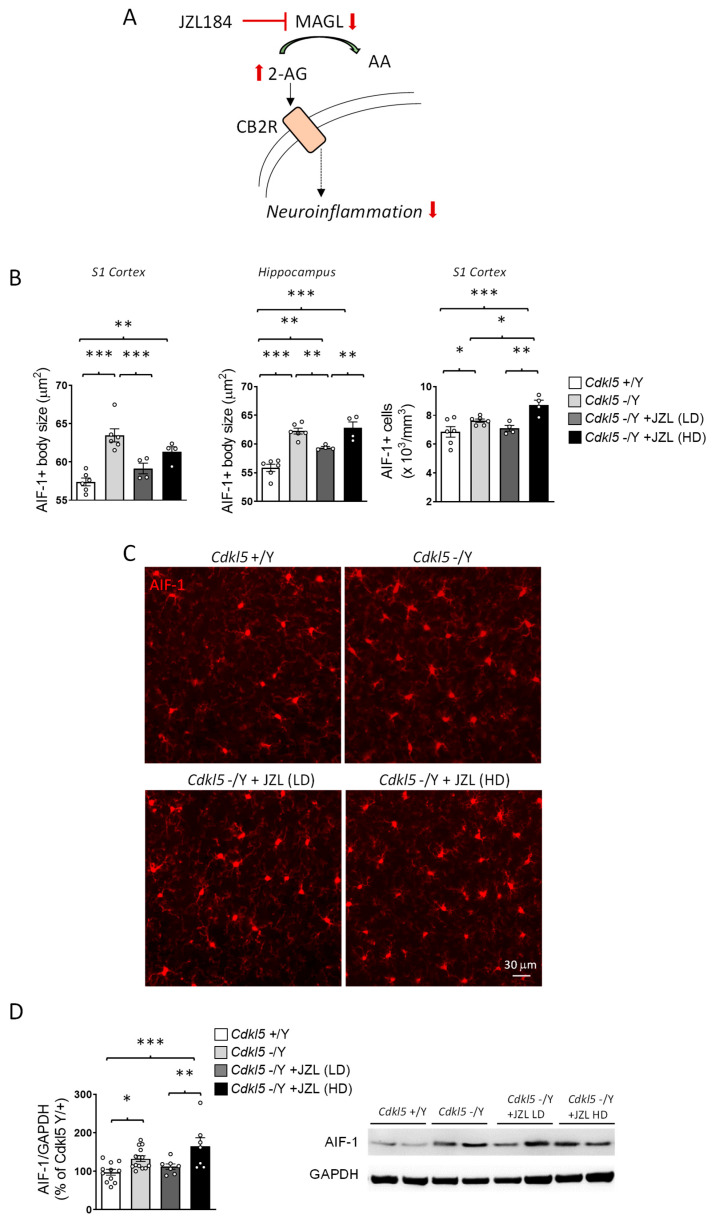
Assessment of microglial activation following six-week JZL184 treatment. (**A**) Schematic overview illustrating the proposed impact of sustained MAGL inhibition by JZL184 on endocannabinoid signaling and downstream cellular responses. Elevated 2-AG levels may engage CB2 receptor–mediated signaling in microglia, thereby contributing to the attenuation of neuroinflammatory responses. (**B**) Mean cell body size of AIF-1-positive cells in the primary somatosensory cortex (S1) (on the left) and in hippocampal sections (in the middle) from vehicle-treated wild-type (+/Y *n* = 6), vehicle-treated *Cdkl5* KO (−/Y *n* = 6) and JZL184-treated *Cdkl5* KO mice (−/Y JZL LD *n* = 4; −/Y JZL HD *n* = 4). Histogram on the right shows microglial cell density in S1. (**C**) Representative immunofluorescence images of AIF-1–positive microglia in the primary somatosensory cortex (S1) across experimental groups. (**D**) Western blot analysis and densitometric quantification of AIF-1 protein levels in cortical tissue extracts from vehicle-treated wild-type (+/Y *n* = 11), vehicle-treated *Cdkl5* KO (−/Y *n* = 14) and JZL184-treated *Cdkl5* KO mice (−/Y JZL LD *n* = 7; −/Y JZL HD *n* = 7). Histogram shows the quantification of AIF-1 levels normalized to GAPDH. The data are shown as the mean ± SEM and are expressed as a percentage relative to the controls. Statistical analyses were performed using one-way ANOVA followed by Fisher’s LSD post hoc test. * *p* < 0.05, ** *p* < 0.01, *** *p* < 0.001.

Since *Cdkl5* KO mice exhibit pronounced microglial activation, and given the established involvement of CB2 receptor signaling in modulating neuroinflammatory responses (Figure 4A) [38,39], we investigated whether sustained MAGL inhibition could counteract this phenotype.

Immunohistochemical analysis of AIF-1–positive microglia confirmed a marked activation state in *Cdkl5* KO mice, characterized by enlarged soma size in both the primary somatosensory cortex (S1) and hippocampus, whereas microglial cell density was quantified only in S1 (Figure 4B,C).

Six-week JZL184 treatment produced dose-dependent and only partial effects on this phenotype. The lower dose attenuated microglial activation, reducing soma size in both cortex and hippocampus and decreasing cortical microglial density (Figure 4B,C). This anti-inflammatory trend was corroborated by Western blot analysis, which showed reduced cortical AIF-1 levels at the lower dose (Figure 4D).

In contrast, the higher dose of JZL184 did not restore microglial soma size, showing only a weak trend in the cortex and no improvement in the hippocampus, and unexpectedly increased microglial density in the cortex (Figure 4B,C). A corresponding increase in cortical AIF-1 protein expression was also detected by Western blot (Figure 4D), indicating enhanced, rather than reduced, microglial reactivity at this dose.

Together, these findings suggest that sustained enhancement of 2-AG signaling can partially mitigate microglial hyperactivation in *Cdkl5* KO mice, but only within a restricted dose range. The observation that the higher dose fails to confer benefit, and may even exacerbate microglial activation, raises the possibility of dose-dependent compensatory or off-target mechanisms that counteract the anti-inflammatory effects of JZL184. This pattern indicates that microglial responses to endocannabinoid modulation are particularly sensitive to 2-AG levels, defining a narrower therapeutic window than that observed for neuronal or synaptic outcomes.

## 3. Discussion

In this study, we investigated whether pharmacological enhancement of endocannabinoid signaling through inhibition of monoacylglycerol lipase (MAGL) could ameliorate behavioral and neurobiological abnormalities in *Cdkl5* knockout (KO) mice, a well-established model of CDKL5 Deficiency Disorder (CDD) [7]. Using a 6-week intermittent treatment with the MAGL inhibitor JZL184 at two doses previously shown to elevate 2-arachidonoylglycerol (2-AG) levels and induce multi-week synaptic adaptations [40,41], we demonstrate robust pharmacodynamic engagement of the target. JZL184 increased plasma 2-AG levels (significantly at the higher dose), reduced MAGL protein abundance, and activated CB1-AKT signaling without inducing CB1 or CB2 receptor downregulation.

Despite this clear molecular efficacy in adult-treated animals, behavioral improvements were domain-specific rather than generalized. Most CDD-relevant phenotypes, including hyperlocomotion, impaired motor coordination, reduced marble burying, and abnormal clasping, were not improved by either dose. However, the higher dose normalized stereotypic vertical jumping and partially restored cue-dependent fear memory, as evidenced by a significant within-group increase in freezing from pre-cue to cue. Although performance did not fully reach wild-type levels, these findings indicate that enhanced 2-AG/CB1 signaling can selectively modulate specific behavioral domains, while remaining insufficient to broadly reverse the established CDD-like phenotype.

Interpretation of behavioral data requires consideration of the sensitivity of the paradigms employed. Passive avoidance performance was unexpectedly poor in wild-type mice, limiting the interpretability of this assay. Prolonged handling and the extended treatment regimen may have induced chronic stress, which is known to impair aversive memory consolidation [42]. Passive avoidance is particularly susceptible to such stress-related effects, potentially masking genotype differences and treatment responses.

In contrast, fear conditioning proved more robust under these experimental conditions. Wild-type mice showed clear cue-dependent freezing, allowing detection of both associative memory deficits in *Cdkl5* KO mice and partial improvement following high-dose JZL184. This dissociation suggests that selective enhancement of 2-AG signaling through MAGL inhibition may not be sufficient to fully normalize complex behavioral phenotypes when administered in adulthood, after CDKL5-dependent developmental alterations are established.

Although both doses of JZL184 produced comparable biochemical evidence of MAGL inhibition and CB1-AKT activation, consistent behavioral improvements emerged only at the higher dose. This dissociation highlights a gap between global molecular target engagement and functional circuit recovery, indicating that effective enhancement of endocannabinoid tone is not, per se, sufficient to normalize behavior once CDKL5-dependent developmental programs are disrupted.

These findings suggest that behavioral modulation depends on circuit-specific functional thresholds rather than on global increases in 2-AG signaling. Circuits regulating motor disinhibition and associative fear learning appear particularly sensitive to higher levels of CB1 engagement, whereas other behavioral domains relevant to CDD remain largely unresponsive in adulthood. Indeed, CB1 receptors are highly expressed in brain regions critically involved in associative fear learning and behavioral inhibition, including the amygdala, hippocampus, and medial prefrontal cortex, where they exert a key modulatory role on synaptic plasticity and memory processes [43]. This pattern supports the notion of a narrow functional window for MAGL inhibition, rather than a broad dose-dependent relationship.

Our structural and cellular analyses provide insight into why behavioral rescue remains limited despite robust pharmacodynamic efficacy. Chronic JZL184 treatment partially corrected abnormalities in dendritic spine maturation in the primary somatosensory cortex (S1), shifting spine morphology toward more mature phenotypes without affecting overall spine density. This is consistent with the established role of 2-AG/CB1 signaling in synaptic stabilization and maintenance rather than in initiating synaptogenic programs [31,44,45].

Similarly, in the hippocampal CA1 region, both doses of JZL184 partially restored neuronal survival, supporting a neuroprotective role for enhanced 2-AG/CB1 signaling in vulnerable circuits. These cellular benefits likely reflect activation of pro-survival pathways downstream of CB1, including AKT phosphorylation [46,47], which promotes cytoskeletal stability, resistance to cellular stress, and synaptic maintenance [48,49]. However, while such mechanisms may stabilize existing circuitry, they are unlikely to re-establish precise wiring, excitatory–inhibitory balance, and long-range connectivity that depend on CDKL5 function during early developmental critical periods.

Heightened neuroinflammation is a prominent pathological feature of CDKL5 deficiency, characterized by sustained microglial activation in the primary somatosensory cortex (S1) and hippocampal regions [50,51]. In this context, MAGL inhibition exerted dose-dependent and bidirectional effects on microglial reactivity. The lower dose of JZL184 attenuated microglial activation, reducing soma size, cortical microglial density, and AIF-1 expression, indicating that the moderate enhancement of 2-AG signaling is sufficient to engage anti-inflammatory pathways.

In contrast, the higher dose failed to confer similar benefits and instead increased cortical microglial density and AIF-1 expression. These pro-inflammatory effects are unlikely to be explained by systemic toxicity or overt cannabinoid receptor downregulation, since body weight, general wellbeing, and CB1/CB2 expression levels remained unchanged; however, functional receptor desensitization cannot be entirely excluded given that receptor activity was not directly assayed. Rather, excessive or sustained elevation of 2-AG may engage compensatory or off-target pathways. For example, MAGL is a major contributor to the brain arachidonate precursor pool for prostaglandin synthesis, and perturbation of 2-AG metabolism can alter AA/eicosanoid homeostasis [45,52]. Alternatively, compensatory changes in other 2-AG hydrolases (e.g., ABHD6/ABHD12) or shifts in downstream COX pathways could modify the balance of pro- vs. anti-inflammatory lipid mediators and thus contribute to pro-inflammatory readouts [52]. In addition, excessive or ectopic CB1 activation at glutamatergic terminals has been associated with dysregulation of excitatory transmission and network homeostasis [53,54], which could further exacerbate circuit-specific inflammatory or functional vulnerabilities. Finally, we note that sustained MAGL blockade can also induce adaptive changes in the endocannabinoid system (including CB1 functional adaptations) that should be borne in mind when interpreting sustained pharmacological manipulations [29].

The dissociation between partial structural rescue and limited behavioral improvement underscores the presence of strong developmental constraints in CDKL5 deficiency. CDKL5 loss disrupts neuronal maturation, synapse formation, and network assembly from early postnatal stages, affecting axonal targeting, interneuron integration, and the establishment of excitatory–inhibitory balance. Consequently, therapeutic interventions initiated in adulthood likely occur after the closure of critical developmental windows. While MAGL inhibition may stabilize existing synaptic structures and promote cellular resilience, it is unlikely to reprogram circuit architecture once aberrant developmental trajectories have been established. This interpretation is further supported by evidence showing that CB1 receptor signaling and MAGL-dependent 2-AG tone play a decisive role during early corticogenesis, where they actively direct neuronal fate specification and circuit assembly, but exert more limited effects once developmental programs are complete [55].

This interpretation is consistent with observations in other neurodevelopmental disorders, including Rett syndrome and Fragile X syndrome, where pharmacological manipulation of the endocannabinoid system produces variable, domain-specific, or transient outcomes [56,57,58]. In CDKL5 models, cannabidiol (CBD) has been reported to exert broader behavioral effects; however, these outcomes likely reflect its pleiotropic pharmacological profile rather than selective enhancement of 2-AG/CB1 signaling alone. Although several molecular targets of CBD, including certain GPCRs, TRP channels, PPAR isoforms, and serotonin receptors, are increasingly regarded as components of an extended endocannabinoid signaling network [15,59], CBD engages these pathways in a multimodal manner distinct from the selective amplification of 2-AG achieved through MAGL inhibition. The more circumscribed effects observed with JZL184 therefore indirectly suggest that selective 2-AG/CB1 modulation engages only a subset of mechanisms potentially relevant to CDKL5 disorder.

A key implication of our findings is that pharmacological elevation of 2-AG via MAGL inhibition should not be considered mechanistically analogous to CBD treatment. Although often grouped under the broader umbrella of cannabinoid-based interventions, CBD does not act as a direct orthosteric CB1 agonist; rather, it functions as a negative allosteric modulator at CB1 receptors and influences endocannabinoid signaling indirectly, while simultaneously engaging multiple additional molecular targets beyond CB1/CB2 receptors [15,59]. By contrast, MAGL inhibition robustly enhances 2-AG levels, a full agonist at CB1 and CB2 receptors, thereby preferentially increasing synaptic CB1 signaling within active neuronal circuits. The limited behavioral rescue observed in adult *Cdkl5* KO mice is therefore consistent with the notion that selective strengthening of 2-AG/CB1 signaling, when administered in adulthood, may be insufficient to overcome the multifactorial and developmentally entrenched alterations underlying CDD. Engagement of additional or complementary signaling pathways, whether within the broader endocannabinoid signaling network or through interacting neuromodulatory systems, may be required to impact complex behavioral phenotypes.

Several limitations should be acknowledged. Treatment was confined to adulthood, precluding evaluation of earlier or prolonged intervention paradigms that might more effectively interact with critical developmental processes. We deliberately initiated treatment at five months of age to determine whether selective MAGL inhibition could reverse already-established neurodevelopmental and behavioral abnormalities, thereby modeling a clinically relevant scenario in which therapeutic interventions are initiated after symptom onset. This approach allowed us to assess the capacity of 2-AG enhancement to act on mature neural circuits and a stable behavioral phenotype, without the confounding influence of ongoing developmental plasticity. Nevertheless, given that CDKL5-dependent alterations emerge early during postnatal brain development, it remains plausible that earlier intervention during critical periods may yield broader or more durable effects. This possibility is further supported by the observation that structural abnormalities, such as dendritic spine immaturity, were only partially rescued under adult treatment conditions, suggesting that interventions during developmental windows of heightened synaptic plasticity might produce more extensive circuit-level normalization. Such developmental-stage interventions, which require distinct dosing strategies and longitudinal safety assessments, were beyond the scope of the present study and will be addressed in future investigations. Seizure activity was not assessed, leaving open the possibility that MAGL inhibition could influence epileptic phenotypes independently of the behavioral domains examined. In addition, our biochemical analyses lack cellular and circuit-level resolution, and direct electrophysiological assessment of synaptic transmission and network activity was not performed. Although structural and molecular findings suggest engagement of synaptic and pro-survival pathways, electrophysiological recordings would provide important mechanistic insight into the functional consequences of MAGL inhibition at the circuit level and represent a critical direction for future investigation. Moreover, although improvements were observed in cue-dependent fear conditioning, amygdala circuits were not directly examined in the present study, limiting the ability to link behavioral changes to region-specific synaptic or inflammatory mechanisms. Finally, the intervention selectively targeted 2-AG degradation through MAGL inhibition without systematically assessing other components of the endocannabinoid system, which constrains interpretation of system-wide conclusions regarding ECS dysregulation in CDKL5 deficiency.

An additional limitation of the present study is that 2-AG levels were quantified only in plasma and were not directly measured in brain tissue or cerebrospinal fluid. Peripheral measurements may not fully reflect the dynamics of central endocannabinoid signaling. Although JZL184 is a well-established brain-penetrant MAGL inhibitor known to elevate central 2-AG levels [29,30], and reduced MAGL protein levels together with increased CB1-dependent AKT phosphorylation were observed in hippocampal extracts in the present study, direct quantification of regional 2-AG levels in cortex and hippocampus would further strengthen the link between pharmacodynamic engagement and behavioral outcomes. Future studies incorporating tissue-specific lipidomic analyses will be important to refine this relationship.

From a translational perspective, these results complement clinical observations in CDD. While CBD-based formulations can reduce seizure burden in subsets of patients [60,61], evidence regarding effects on cognition, behavior, and overall quality of life remains limited, heterogeneous, and largely derived from mixed-etiology or uncontrolled studies, with scarce data specific to CDD [12,23]. Our findings indicate that, under the present experimental conditions, selective enhancement of 2-AG signaling was associated with more circumscribed effects than those reported for CBD in clinical observations. Notably, specific behavioral domains (such as motor disinhibition and associative fear learning) and related structural features (including dendritic spine maturation in the primary somatosensory cortex and CA1 neuronal survival) appeared responsive to selective 2-AG enhancement via MAGL inhibition, whereas broader alterations remained largely unaffected.

## 4. Materials and Methods

### 4.1. Mouse Colony

The mice used in this study belonged to the *Cdkl5* knockout (KO) strain on a C57BL/6N background originally generated as previously described [7] and subsequently backcrossed for three generations onto a C57BL/6J background. For the present experiments, mice were obtained by crossing *Cdkl5* heterozygous females (+/−) with wild-type males (+/Y). Genotyping was performed by PCR analysis of genomic DNA as previously reported [7].

Littermate controls were used for all experiments. The day of birth was designated as postnatal day 0 (P0), and animals within 24 h of birth were considered P1. After weaning (P21–P23), mice were group-housed (3–5 animals per cage) in a temperature-controlled environment (23 °C) under a 12 h light/dark cycle, with ad libitum access to food and water. Animal health and welfare were monitored daily by the institutional veterinary service. Not all animals were included in every experimental analysis due to tissue availability and assay-specific requirements. Exact sample sizes are reported in the corresponding figure legends. All experimental procedures were conducted in accordance with Italian and European Community regulations for the use of experimental animals and were approved by the National Bioethical Committee (authorization number: 375/2024-PR, protocol reference AEDB0.41), approved on 23 April 2024.

Experiments were carried out on a total of 39 adult male mice, including *Cdkl5* +/Y (*n* = 12) and *Cdkl5* −/Y (*n* = 27) animals. Only male mice were used to ensure phenotypic consistency and to avoid variability associated with X-linked mosaicism in heterozygous females.

### 4.2. JZL184 Treatment

Starting at five months of age (adult stage), mice were treated intraperitoneally (i.p.) with either vehicle (5% DMSO, 40% PEG300, 5% Tween-80, and 50% ddH_2_O) or the monoacylglycerol lipase (MAGL) inhibitor JZL184 (10 or 20 mg/kg; Selleckchem, Munich, Germany) formulated in the same vehicle. Treatments were administered on an alternate-day schedule (three times per week: Monday, Wednesday, and Friday) for six consecutive weeks (Figure 1A).

The two doses were selected based on previous studies. The lower dose (10 mg/kg; LD) has been shown to produce robust MAGL inhibition and elevation of 2-AG levels with partial engagement of CB1-dependent tolerance mechanisms during repeated exposure [30]. The higher dose (20 mg/kg; HD) was chosen to achieve stronger CB1-mediated signaling and to test whether a more pronounced and sustained increase in 2-AG could impact the structural and behavioral abnormalities associated with CDKL5 deficiency.

An intermittent dosing schedule was adopted to maintain sustained elevation of 2-AG while minimizing the risk of CB1 receptor desensitization, downregulation, and behavioral tolerance reported with daily high-dose MAGL inhibition [29]. Previous studies have demonstrated that multi-week MAGL inhibition (approximately 3–4 weeks) is sufficient to induce durable synaptic and behavioral adaptations in mouse models. In particular, 6-week JZL184 administration improved hippocampal synaptic plasticity and cognitive performance in Ts65Dn mice following multi-week treatment [40]. Moreover, sustained MAGL blockade has been shown to induce functional adaptations of the endocannabinoid system, including CB1 receptor tolerance after prolonged 2-AG elevation [29]. Accordingly, a 6-week treatment duration was selected to fall within, and modestly extend, this established multi-week window, allowing sufficient time for potential circuit-level remodeling while limiting the risk of receptor desensitization.

Body weight was monitored throughout the treatment period on injection days. Behavioral testing was initiated at the end of the fourth week of treatment and continued during the final two weeks of the regimen. The behavioral battery was arranged from the least invasive to the most demanding paradigms to minimize cumulative stress and potential carry-over effects. On the day following completion of behavioral testing, mice were sacrificed and brain tissues were collected for histological and Western blot analyses.

### 4.3. Quantification of 2-Arachidonoylglycerol (2-AG) in Mouse Plasma

2-Arachidonoylglycerol (2-AG) was purchased from Sigma-Aldrich (St. Louis, MO, USA), and the deuterated internal standard 2-arachidonoylglycerol-d8 (2-AG-d8) was obtained from Cayman Chemical (Ann Arbor, MI, USA). Acetonitrile and methanol (HPLC–MS grade) were supplied by VWR (Radnor, PA, USA), and formic acid (LC–MS grade) was purchased from Carlo Erba (Milan, Italy). All other reagents were of analytical grade.

Plasma 2-AG levels were quantified using a protein precipitation-based extraction protocol optimized to limit endocannabinoid degradation and adsorption to plastic surfaces, adapted with minor modifications from a previously validated method [62]. Mouse plasma samples were processed together with pooled quality control (QC) samples, prepared by combining equal aliquots of all study samples and used to monitor analytical reproducibility throughout the sequence.

Briefly, 10 µL of plasma was transferred into 2 mL low-binding polypropylene tubes (Eppendorf LoBind, Hamburg, Germany). Methanol was pre-chilled at −20 °C, and 100 µL was added to each sample, followed by 90 µL of acetonitrile and 10 µL of internal standard solution (2-AG-d8, 500 ng/mL in acetonitrile). Samples were vortex-mixed for 2 min, agitated at 5000 rpm for 10 min at 4 °C, and centrifuged to induce protein precipitation. Subsequently, 185 µL of supernatant was transferred to new low-binding tubes and evaporated to dryness using a Savant SpeedVac SPD120 vacuum concentrator (Thermo Fisher Scientific, Waltham, MA, USA) equipped with a Savant RVT5105 refrigerated vapor trap (Thermo Fisher Scientific, Waltham, MA, USA). Dried extracts were reconstituted in 100 µL of acetonitrile:water (70:30, *v*/*v*) containing 0.1% formic acid, vortex-mixed, sonicated in an ice-cooled ultrasonic bath for 2 min, and centrifuged at 13,000 rpm for 10 min at 4 °C. The final supernatants were transferred to autosampler vials for LC–MS/MS analysis.

Chromatographic separation was performed on a Nexera X2 UHPLC system (Shimadzu, Kyoto, Japan) coupled to a QTRAP 4500 triple quadrupole mass spectrometer (SCIEX, Framingham, MA, USA) equipped with an electrospray ionization (ESI) source. Analytes were separated on a Kinetex C18 column (2.6 µm, 100 Å, 30 × 4.6 mm i.d.; Phenomenex, Torrance, CA, USA). The mobile phases consisted of water with 0.1% formic acid (A) and acetonitrile with 0.1% formic acid (B). The column temperature was maintained at 30 °C, the flow rate was set at 0.6 mL min^−1^, and the injection volume was 10 µL. The gradient program was: 30% B at 0.0 min, increased to 70% B at 2.89 min, to 90% B at 3.50 min, and to 99.8% B at 12.50 min, followed by re-equilibration to 30% B until 15.05 min (total run time 15 min).

Mass spectrometric detection was carried out in positive ion mode using multiple reaction monitoring (MRM). 2-AG was monitored using the transitions *m*/*z* 379 → 287 (quantifier) and *m*/*z* 379 → 269 (qualifier), while 2-AG-d8 was monitored at *m*/*z* 387 → 294. Two partially resolved chromatographic peaks corresponding to the 1- and 2-arachidonoylglycerol isomers were detected at retention times of approximately 4.59 and 4.65 min, respectively; quantification was performed by integrating the combined peak areas of both isomers.

Plasma 2-AG concentrations were determined using a standard addition approach, based on a six-point calibration curve (5–100 ng/mL) generated by spiking pooled QC plasma samples. Calibration curves were fitted using weighted (1/x^2^) linear regression, and calibration samples were processed identically to study samples. All samples were randomized prior to injection, and quantitative data are reported as the mean of duplicate analyses.

Throughout the analytical sequence, pooled QC samples were injected repeatedly as technical replicates (reinjections of the same extract) and biological replicates (independent extractions) to assess method reproducibility. Solvent and procedural blanks were included to monitor carryover and background contamination. Eight QC injections were performed at the beginning of the sequence to allow column conditioning and system stabilization.

### 4.4. Behavioral Assays

Behavioral testing was initiated at the end of the fourth week of the 6-week JZL184 treatment regimen and continued during the final two weeks of treatment. Tests were administered in a fixed order, from the least to the most stressful paradigms, to minimize cumulative stress and potential carry-over effects. Animals were allowed a 1-day recovery period between consecutive tests. All behavioral experiments and data analyses were conducted by investigators blinded to genotype and treatment. Prior to each test, mice were habituated to the testing room for at least 1 h. All behavioral assessments were performed at the same time of day to minimize variability related to circadian rhythms.

#### 4.4.1. Marble Burying Test

Mice were individually placed in a home-cage-like arena containing 5 cm of unscented bedding and 20 glass marbles (14.3 mm diameter) arranged in a 4 × 5 grid. Animals were left undisturbed for 30 min, after which the number of marbles buried at least two-thirds was counted.

#### 4.4.2. Hindlimb Clasping

Mice were suspended by the tail for 2 min, and hindlimb clasping time was quantified from video recordings. A clasping event was defined as retraction of one or both hindlimbs toward the abdomen and midline.

#### 4.4.3. Accelerating Rotarod

Mice were briefly trained on a rotarod apparatus (Ugo Basile, Gemonio, Italy) at a constant speed of 5 rpm for 30 s. Thirty minutes later, testing was performed using a linear acceleration protocol (5–35 rpm over 270 s, followed by 30 s at maximum speed). Four trials were conducted with a 1 h inter-trial interval. Both latency to fall and number of passive rotations (defined as rotations during which the mouse passively rotates without coordinated locomotion) were recorded.

#### 4.4.4. Open Field Test

Spontaneous locomotor activity was assessed in a 50 × 50 cm square arena over a 20 min session. Total distance traveled and average velocity were analyzed using EthoVision XT 15 software (Noldus Information Technology, Wageningen, The Netherlands). Stereotypic vertical jumps were manually identified and counted during the test by two independent observers blinded to genotype and treatment, based on clearly distinguishable vertical escape-like movements. The arena was cleaned with 70% ethanol between sessions.

#### 4.4.5. Passive Avoidance

The passive avoidance task was performed using a two-compartment apparatus (Ugo Basile, Gemonio, Italy). During training, mice received a 0.4 mA foot shock for 3 s upon entry into the dark compartment. Retention was tested 24 h later, and latency to re-enter the dark compartment was recorded up to a cutoff time of 360 s.

#### 4.4.6. Fear Conditioning

Fear conditioning was conducted in a sound-attenuated chamber (30 × 24 × 21 cm; Ugo Basile, Gemonio, Italy). On day 1, mice were allowed to explore for 2 min, followed by two pairings of a 20 s auditory cue (85 dB, 2 kHz) co-terminating with a 0.75 mA foot shock (last 2 s). On day 2, contextual memory was assessed, followed by a cue test in a novel chamber. Freezing behavior was automatically quantified using EthoVision XT 15 software (Noldus Information Technology, Wageningen, The Netherlands).

#### 4.4.7. Von Frey Filament Test

Mechanical sensitivity was assessed using von Frey filaments (Ugo Basile, Gemonio, Italy). Mice were acclimated for 1 h in acrylic chambers positioned on a wire-mesh floor. A single calibrated filament was applied perpendicularly to the plantar surface of each hind paw with increasing force until a withdrawal response was observed. Withdrawal latency and force threshold were calculated as the mean of three measurements per paw.

### 4.5. Histology and Immunohistochemistry

Mice were deeply anesthetized with 2% isoflurane in oxygen and euthanized by cervical dislocation. Brains were rapidly removed and bisected along the midline. Left hemispheres were processed for Golgi staining or snap-frozen for Western blot analysis. Right hemispheres were immersion-fixed in 4% paraformaldehyde (PFA) in 0.1 M PBS for 48 h at 4 °C, cryoprotected in 20% sucrose, frozen on dry ice, and stored at −80 °C. Coronal sections (30 µm) were cut using a freezing microtome (Microm GmbH, Neuss, Germany) and stored in antifreeze solution (30% glycerol, 30% ethylene glycol, 0.02% sodium azide in 0.1 M PBS) until processing.

#### 4.5.1. Immunofluorescence Staining

One out of every eight free-floating coronal sections spanning the hippocampal formation and the overlying dorsal cortex was processed for immunofluorescence analysis. Sections encompassed rostrocaudal levels extending approximately from −1.2 mm to −3.8 mm relative to bregma, according to the Paxinos and Franklin mouse brain atlas (2nd edition). Free-floating sections were incubated overnight at 4 °C with a rabbit polyclonal anti-AIF-1 antibody (1:300; Thermo Fisher Scientific, Waltham, MA, USA). On the following day, sections were incubated for 2 h at room temperature with an Alexa Fluor™ 555–conjugated anti-rabbit IgG (H+L) secondary antibody (1:200; Invitrogen, Thermo Fisher Scientific, Waltham, MA, USA). Detailed information, including catalog numbers and host species for all antibodies used, is provided in Table S1. Primary and secondary antibodies were diluted in 1% Bovine Serum Albumin (BSA) prepared in phosphate-buffered saline (PBS) containing 0.1% Triton X-100. Sections were counterstained and mounted with DAPI (40,6-diamidino-2-phenylindole)-Fluoromount-G^®^ (SouthernBiotech, Birmingham, AL, USA). Fluorescent images were acquired using an Eclipse TE 2000-S microscope equipped with a DS-Qi2 digital SLR camera (Nikon Instruments, Tokyo, Japan).

#### 4.5.2. Golgi Staining

Left hemispheres were Golgi-stained using the FD Rapid Golgi Stain^TM^ Kit (FD Neuro Technologies, Columbia, MD, USA). Briefly, hemispheres were immersed in the impregnation solution containing mercuric chloride, potassium dichromate, and potassium chromate, and stored at room temperature in the dark for 3 weeks. Hemispheres were then cut with a cryostat (Histo-Line Laboratories, Pantigliate, Italy) into 100-µm-thick coronal sections, which were directly mounted onto Superfrost^®^ Plus Microscope Slides (Thermo Fisher Scientific, Waltham, MA, USA) and air-dried at room temperature for 1 day. After drying, sections were rinsed with distilled water, stained in the developing solution of FD Rapid Golgi Stain^TM^ Kit (FD NeuroTechnologies, Columbia, MD, USA), and coverslipped with DPX mounting medium (Sigma-Aldrich, Saint Louis, MO, USA). A light microscope (Leica Microsystems, Wetzlar, Germany) equipped with motorized stage, focus control system, and color digital camera (Coolsnap-Pro; Media Cybernetics, Rockville, MD, USA) were used to acquire bright field images.

### 4.6. Measurements

#### 4.6.1. Cell Density

The density of DAPI-positive nuclei was quantified in the CA1 pyramidal layer across coronal sections sampled every sixth section, spanning the rostrocaudal extent of the dorsal to intermediate hippocampus (approximately −1.2 to −3.8 mm from bregma, according to Paxinos and Franklin). DAPI-positive nuclei within the CA1 pyramidal layer were manually counted and expressed as cells/mm^3^, and were used as a proxy for neuronal density given the highly homogeneous neuronal composition of this region.

The number of AIF-1-positive cells was quantified exclusively in the primary somatosensory cortex (S1), across all cortical layers of the dorsal cortical region overlying the hippocampus. Both AIF-1- and DAPI-positive cells were manually counted using the same Image-Pro Plus software (version 4.5; Media Cybernetics, Silver Spring, MD, USA) and expressed as cells/mm^3^.

#### 4.6.2. Morphometric Microglial Cell Analysis

Morphometric analyses were performed on 20× magnification images of AIF-1-stained sections acquired from the CA1 region of the hippocampus and from the primary somatosensory cortex (S1), across all cortical layers within the dorsal cortical mantle overlying the hippocampus (−1.2 to −3.8 mm from bregma). Microglial cell body size was manually delineated using the measurement function of Image-Pro Plus software (version 4.5; Media Cybernetics, Rockville, MD, USA) and expressed in μm^2^. Approximately 120 microglial cells were analyzed per sample.

#### 4.6.3. Dendritic Spine Number and Morphology

In Golgi-stained sections, dendritic spines were analyzed on basal dendrites of pyramidal neurons located in layers II/III of the primary somatosensory cortex (S1), in the dorsal cortical region overlying the hippocampus (−1.2 to −3.8 mm from bregma). Dendritic spines were visualized using a 100× oil immersion objective lens. Dendritic spine density was measured by manually counting the number of dendritic spines on the basal dendrites of cortical pyramidal neurons and was expressed as the total number of spines per 10 μm. Based on their morphology, dendritic spines can be divided into two different categories that reflect their state of maturation: immature spines (filopodium-like, thin- and stubby-shaped) and mature spines (mushroom- and cup-shaped). The number of mature spines was counted and expressed as a percentage. About 100–150 spines from 15 to 20 dendrites, derived from 10 to 20 neurons, were analyzed per condition.

### 4.7. Western Blotting

Tissue samples from the hippocampus of vehicle-treated *Cdkl5* +/Y and *Cdkl5* −/Y mice and of JZL184-treated *Cdkl5* −/Y mice were lysed in a ice-cold RIPA buffer (50 mM Tris–HCl, pH 7.4, 150 mM NaCl, 1% Triton-X100, 0.5% sodium deoxycholate, 0.1% SDS) supplemented with 1 mM PMSF, as well as with 1% protease and phosphatase inhibitor cocktail (Sigma-Aldrich, Saint Louis, MO, USA). Protein concentration for tissue extracts was determined using the Bradford method [63]. Equivalent amounts of protein (50 µg) were subjected to electrophoresis on a BoltTM 4–12% Bis-Tris Plus gel (Life Technologies Corporation—Thermo Fisher Scientific Inc., Waltham, MA, USA) and transferred to a Hybond ECL nitrocellulose membrane (GE Healthcare Bio-Science, Piscataway, NJ, USA). The following primary antibodies were used: mouse monoclonal anti-MAGL (1:1000; Santa Cruz Biotechnology, Dallas, TX, USA), mouse monoclonal anti-CBR1 (1:1000; Santa Cruz Biotechnology, Dallas, TX, USA), mouse monoclonal anti-CBR2 (1:1000; Santa Cruz Biotechnology, Dallas, TX, USA), rabbit polyclonal anti-phospho-Akt (Ser473; 1:1000; Cell Signaling Technology, Danvers, MA, USA), rabbit polyclonal anti-Akt (1:1000; Cell Signaling Technology, Danvers, MA, USA), rabbit polyclonal anti-AIF-1 antibody (1:1000;Thermo Fisher), rabbit polyclonal anti-GAPDH (1:5000; Sigma-Aldrich, Saint Louis, MO, USA), and mouse monoclonal anti-Vinculin (1:1000, Santa Cruz Biotechnology, Dallas, TX, USA). An HRP-conjugated goat anti-rabbit IgG secondary antibody (1:5000; Jackson ImmunoResearch Laboratories, West Grove, PA, USA) and an HRP-conjugated goat anti-mouse IgG secondary antibody (1:5000; Jackson ImmunoResearch Laboratories, West Grove, PA, USA) were used. Detailed information, including catalog numbers and host species for all antibodies used, is provided in Table S1. Densitometric analysis of digitized Western blot images was performed using ChemiDocTM MP Imaging System equipped with Image Lab Touch Software (version 3.0.1, Bio-Rad, Hercules, CA, USA). This software automatically highlights any saturated pixels of Western blot images in red. Images acquired with exposure times that generated protein signals out of a linear range were not considered for quantification. Western blot analyses were performed on protein extracts of multiple samples per experimental group (three to six for animals). Repeated measurements of the same samples were performed by running from two to four independent gels. The signal of one sample (internal control) was used to perform a relative analysis of the antigen expression of each sample on the same gel. We considered the control signal as 100 and assigned a value to the other sample as a percentage of the control. Data analysis was performed by averaging the signals obtained in two to five gels for each individual sample.

### 4.8. Statistical Analysis

Statistical analysis was performed using GraphPad Prism 8.0.1 (GraphPad Software, Boston, MA, USA). Data are expressed as means ± SEM. Data distribution was assessed for normality prior to statistical testing. Comparisons were performed using Student’s t-test, one-way ANOVA, or two-way ANOVA (for repeated time-bin analyses), followed by appropriate post hoc tests as specified in the figure legends. A probability level of *p* < 0.05 was considered statistically significant. Complete statistical details, including F-values, degrees of freedom, and exact *p*-values for all analyses, are reported in Supplementary Table S2.

## 5. Conclusions

Together, these findings indicate that selective enhancement of 2-AG signaling via MAGL inhibition was insufficient in adult-treated mice to reverse established neurodevelopmental deficits in CDKL5 deficiency. Rather, they define a narrow therapeutic window in which targeted ECS modulation may yield circuit-specific benefits, emphasizing the need for developmentally informed, multimodal strategies targeting synaptic maturation, network organization, and neuroimmune balance. Future strategies may require the combination of ECS modulation with interventions aimed at enhancing developmental plasticity or targeting complementary molecular pathways.

## Data Availability

The original contributions presented in this study are included in the article/Supplementary Material. Further inquiries can be directed to the corresponding author.

## Supplementary Materials

The following supporting information can be downloaded at: https://www.mdpi.com/article/10.3390/ijms27062773/s1.
