# Supplementary material for: Endocannabinoid Enhancement via MAGL Inhibition in CDKL5 Deficiency: Selective Cellular Benefits and Domain-Specific Functional Effects in Adult Cdkl5 KO Mice
Source: Int J Mol Sci. 2026 Mar 19;27(6):2773. doi: 10.3390/ijms27062773 (PMC13026865; doi:10.3390/ijms27062773)
Supplement: Supplementary file 1 [file ijms-27-02773-s001.zip › ijms-4177430-supplementary.pdf]

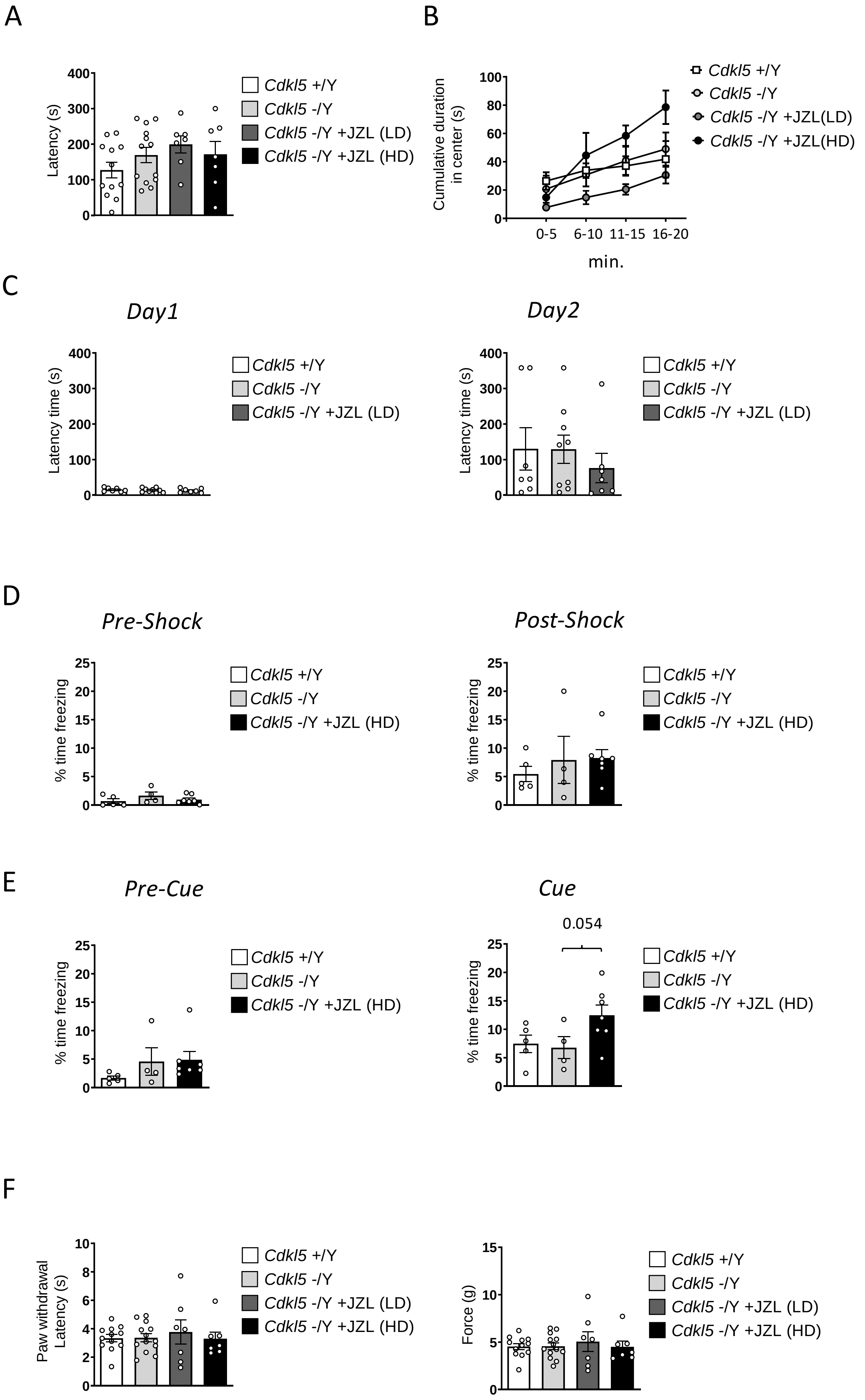
A
B
C
D
E
F
Latency (s)
Cdkl5 +/Y
Cdkl5 -/Y
Cdkl5 -/Y +JZL (LD)
Cdkl5 -/Y +JZL (HD)
Cumulative duration in center (s)
0-5
6-10
11-15
16-20
min.
Day1
Day2
Latency time (s)
Pre-Shock
Post-Shock
% time freezing
Pre-Cue
Cue
0.054
Paw withdrawal Latency (s)
Force (g)

Figure S1. Additional behavioral and sensory assessments. (**A**) Rotarod performance expressed as latency to fall from an accelerating rotating rod in vehicle-treated wild-type mice (+/Y, n = 11), vehicle-treated *Cdkl5* KO mice (−/Y, n = 13), and JZL184-treated *Cdkl5* KO mice receiving a low dose (−/Y JZL LD, n = 6) or a high dose (−/Y JZL HD, n = 7). Mice underwent four trials separated by a 1 h intertrial interval. The graph shows the mean latency to fall averaged across trials. (**B**) Cumulative time spent in the center of the open-field arena (20 min session) in vehicle-treated wild-type mice (+/Y, n = 12), vehicle-treated *Cdkl5* KO mice (−/Y, n = 13), and JZL184-treated *Cdkl5* KO mice (−/Y JZL LD, n = 6; −/Y JZL HD, n = 7). (**C**) Passive avoidance performance during the retention test, expressed as step-through latency on the first (left) and second (right) test days, in vehicle-treated wild-type mice (+/Y *n* = 5), vehicle-treated *Cdkl5* KO mice (−/Y *n* = 4), and *Cdkl5* KO mice treated with a low dose of JZL184 (−/Y JZL LD *n* = 7, 10 mg/kg). (**D**) Freezing behavior during the fear conditioning training session, measured during the pre-shock and post-shock phases, in vehicle-treated wild-type mice (+/Y *n* = 5), vehicle-treated *Cdkl5* KO mice (−/Y *n* = 4), and *Cdkl5* KO mice treated with a high dose of JZL184 (−/Y JZL HD *n* = 7, 20 mg/kg). (**E**) Freezing behavior measured during the pre-cue and cue phases of the fear conditioning recall test across experimental groups described in panel B. (**F**) Mechanical nociceptive thresholds assessed using the von Frey filament test in vehicle-treated wild-type mice (+/Y, n = 11), vehicle-treated *Cdkl5* KO mice (−/Y, n = 13), and *Cdkl5* KO mice treated with either a low dose (LD, 10 mg/kg, n = 7) or a high dose (HD, 20 mg/kg, n = 7) of JZL184. Mechanical sensitivity was evaluated by measuring withdrawal latency (left panel) and force threshold (g) required to elicit paw withdrawal (right panel). Data are expressed as mean ± SEM. Statistical analyses were performed using one-way ANOVA for panels A and C–F, and two-way ANOVA for panel B, followed by Fisher's LSD post hoc test.

Table S1. Detailed information for all antibodies used.

**Antibody against**	**Description**	**Diluition**	**Product nr and Manufacturer**	**Use ***
phospho-AKT- Ser473	Rabbit polyclonal	1:1000	4060, Cell Signaling Technology, Danvers, MA, USA	WB
AKT	Rabbit polyclonal	1:1000	4691, Cell Signaling Technology, Danvers, MA, USA	WB
AIF-1	Rabbit polyclonal	1:1000	PA5-21274, Invitrogen, Thermo Fisher Scientific, Waltham, MA, USA	WB
		1:300		IF
Monoglyceride Lipase (C-11)	Mouse monoclonal	1:1000	398942, Santa Cruz Biotechnology, Dallas, TX, USA	WB
CB1/Cannabinoid Receptor 1/CNR1 (C-11)	Mouse monoclonal	1:1000	518035, Santa Cruz Biotechnology, Dallas, TX, USA	WB
CB2/Cannabinoid Receptor 2/CNR2 (3C7)	Mouse monoclonal	1:1000	293188, Santa Cruz Biotechnology, Dallas, TX, USA	WB
Vinculin (7F9)	Mouse monoclonal	1:1000	73614, Santa Cruz Biotechnology, Dallas, TX, USA	WB
GAPDH	Rabbit polyclonal	1:5000	G9545, Sigma-Aldrich, Saint Louis, MO, USA	WB
**Secondary antibodies**				
**Antibody**	**Conjugate**	**Diluition**	**Product nr and Manufacturer**	**Use***
Goat Anti-Rabbit IgG	HRP	1:5000	111-035-003, Jackson ImmunoResearch Laboratories, West Grove, PA, USA	WB
Goat anti-mouse IgG	HRP	1:5000	115-035-003, Jackson ImmunoResearch Laboratories, West Grove, PA, USA	WB
Goay anti-rabbit IgG (H+L)	Alexa Fluor™ 555	1:200	A21428, Invitrogen, Thermo Fisher Scientific, Waltham, MA, USA	IF

*WB, western blot; IF, immunofluorescent staining

Table S2. Detailed statistical results for all experimental analyses, including F-values, degrees of freedom, and exact p-values for all analyses

Figure	*Test*	*F (DFn, DFd)*	*P-Value*
1B	one-way ANOVA	F (3, 35) = 0.2838	*p* = 0.8368
1C	one-way ANOVA	F (3, 35) = 5.889	*p* = 0.0023
1D	one-way ANOVA	F (3, 35) = 1.801	*p* = 0.165
1E	one-way ANOVA	F (3, 33) = 2.424	*p* = 0.0832
1F	one-way ANOVA	F (3, 35) = 2.251	*p* = 0.0996
1G	two-way ANOVA	F (3, 140) = 1.110 (genotype-treatment)	*p* = 0.3472
1H	two-way ANOVA	F (3, 140) = 1.320 (genotype-treatment)	*p* = 0.2703
1I	one-way ANOVA	F (3, 34) = 2.305 (genotype-treatment)	*p* = 0.0943
1J	two-way ANOVA	F (2, 26) = 4.076 (time)	*p* = 0.0289
		F (1, 26) = 19.77 (genotype-treatment)	*p* = 0.0001
S1A	one-way ANOVA	F (3, 35) = 1.363	*p* = 0.27
S1B	two-way ANOVA	F (3, 140) = 5.110 (genotype-treatment)	*p* = 0.0022
S1C (Day 1)	one-way ANOVA	F (2, 20) = 0.3551	*p* = 0.7054
S1C (Day 2)	one-way ANOVA	F (2, 20) = 0.4192	*p* = 0.6632
S1D (Pre-)	one-way ANOVA	F (2, 13) = 1.151	*p* = 0.3466
S1D (Post-)	one-way ANOVA	F (2, 13) = 0.4781	*p* = 0.6304
S1E (Pre-Cue)	one-way ANOVA	F (2, 13) = 1.292	*p* = 0.3078
S1E (Cue)	one-way ANOVA	F (2, 13) = 3.020	*p* = 0.0837

Figure		*Test*		*F (DFn, DFd)*	*P-Value*
S1F (Latency)		one-way ANOVA		F (3, 35) = 0.2070	*p* = 0.8909
S1F (Force)		one-way ANOVA		F (3, 35) = 0.1986	*p* = 0.8967
2B		one-way ANOVA		F (3, 34) = 3.533	*p* = 0.0249
2D	MAGL	one-way ANOVA		F (3, 30) = 8.892	*p* = 0.0002
	PAKT/AKT	one-way ANOVA		F (3, 22) = 4.413	*p* = 0.0142
	AKT/GAPDH	one-way ANOVA		F (3, 22) = 1.552	*p* = 0.2293
	CB1R	one-way ANOVA		F (3, 30) = 1.266	*p* = 0.3037
	CB2R	one-way ANOVA		F (3, 21) = 0.9971	*p* = 0.4135
3A		one-way ANOVA		F (3, 20) = 0.6298	*p* = 0.6043
3B		one-way ANOVA	Filipodium	F (3, 20) = 34.30	*p* < 0.0001
			Stubby	F (3, 20) = 0,6306	*p* = 0.6038
			Mushroom	F (3, 20) = 39.87	*p* < 0.0001
			Cup	F (3, 20) = 1.202	*p* = 0.3346
3D		one-way ANOVA		F (3, 16) = 15.28	*p* < 0.0001
4B	Cortex (body size)	one-way ANOVA		F (3, 16) = 15.78	*p* < 0.0001
	Hippo (body size)	one-way ANOVA		F (3, 16) = 26.53	*p* < 0.0001
	Cortex (cell density)	one-way ANOVA		F (3, 16) = 7.911	*p* = 0.0018
4D		one-way ANOVA		F (3, 35) = 6.278	*p* = 0.0016
